# Endovascular Treatment of Hepatic Artery Pseudoaneurysm after Pancreaticoduodenectomy: A Literature Review

**DOI:** 10.3390/life14080920

**Published:** 2024-07-24

**Authors:** Beata Jabłońska, Sławomir Mrowiec

**Affiliations:** Department of Digestive Tract Surgery, Medical University of Silesia, 40-752 Katowice, Poland; mrowasm@poczta.onet.pl

**Keywords:** hepatic artery, pseudoaneurysm, pancreaticoduodenectomy, endovascular treatment, embolization, stent graft

## Abstract

Pancreaticoduodenectomy (PD) is a complex surgical procedure performed in patients with periampullary tumors located within the pancreatic head, the papilla of Vater, the distal common bile duct, and the duodenum. In advanced tumors, the operative technique involves the need for dissection and divestment of the arteries located within the pancreaticoduodenal field, including the common hepatic artery (CHA) and the proper hepatic artery (PHA) and its branches. The second most important cause of post-PD visceral aneurysms is irritation of the peri-pancreatic arterial wall by pancreatic juice in a postoperative pancreatic fistula (POPF). Hepatic artery pseudoaneurysm (HAP) is a very dangerous condition because it is usually asymptomatic, but it is a rare and potentially lethal pathology because of the high risk of its rupture. Therefore, HAP requires treatment. Currently, selective celiac angiography is the gold-standard diagnostic and therapeutic management for postoperative bleeding and pseudoaneurysm in patients following PD. Open surgery and less invasive endovascular treatment are performed in patients with HAP. Endovascular treatment involves transarterial embolization (TAE) and stent graft implantation. The choice of treatment method depends on the general and local conditions, such as the patient’s hemodynamic stability and arterial anatomy. In patients in whom preservation of the flow within the hepatic artery (to prevent hepatic ischemia complications such as liver infarction, abscess, or failure) is needed, stent graft implantation is the treatment of choice. This article focuses on a review of two common methods for endovascular HAP treatment. In addition, risk factors and diagnostic tools have been described.

## 1. Introduction

Pancreaticoduodenectomy (PD) is a complex surgical procedure performed in patients with periampullary tumors located within the pancreatic head, the papilla of Vater, the distal common bile duct, and the duodenum [1,2]. In cases of advanced large tumors, the operative technique involves the need for dissection and divestment of the arteries located within the pancreaticoduodenal field, including the common hepatic artery (CHA) and proper hepatic artery (PHA) and its branches, as well as the celiac trunk (CT) and the superior mesenteric artery (SMA), which may be associated with exposure of the vascular wall and a higher risk of tissue irritation [2,3]. The second most important cause of post-PD visceral aneurysms is irritation of the peri-pancreatic arterial wall by pancreatic juice in a postoperative pancreatic fistula (POPF). It is known that delayed post-pancreatectomy hemorrhage (PPH), as one of the most severe vascular adverse events, is usually caused by the rupture of a pseudoaneurysm [3]. Post-PD pseudoaneurysms are an uncommon complication, but they are related to life-threatening outcomes in up to 50% of cases due to the development of postoperative bleeding [2].

Hepatic artery aneurysms (HAAs) are the second most common type of splanchnic aneurysms, while splenic artery aneurysms (SAAs) are the most common type [4,5]. HAAs constitute approximately 20% of visceral aneurysms. They are reported in 0.002–0.4% of the general population. The incidence of hepatic artery pseudoaneurysm (HAPs) is much lower. These aneurysms constitute approximately 20% of HAAs. HAPs can involve intrahepatic or extrahepatic artery portions, while the extrahepatic part is involved in 80% of HAAs [4].

The first case of HAP was reported by James Wilson in 1809 during an autopsy after its rupture [6]. HAP is a very dangerous condition because it is usually asymptomatic, but it is a rare and potentially lethal pathology because of the high risk of its rupture [7,8,9]. The mortality related to its rupture is up to 70% [10]. Most frequently, HAP is a result of blunt trauma, iatrogenic injury, and inflammatory processes within the abdominal cavity. Less frequently, HAP can be caused by arterial dissection during surgical procedures performed within the epigastrium, i.e., PD [7]. HAPs following pancreatic, biliary, liver, pancreatic, and gastric procedures, penetrating or blunt abdominal trauma, recent orthotopic liver transplants, liver biopsy, infections, and inflammatory pathologies such as acute pancreatitis and due to atherosclerosis have been reported in the world literature [2,3,4,5,6,7,8,9,10].

In 1903, Kehr described the first successful ligation of an HAA [5,11]. Since then, HAA management, including various surgical techniques, has evolved. The appropriate HAA treatment depends primarily on aneurysm location, presence of collateral flow, and operative risk, as well as the patient’s clinical status. Different open surgical treatment techniques for HAA are distinguished, such as ligation, excision, venous grafting, synthetic grafting, and liver resection [5,10,11,12,13,14,15,16,17]. Intrahepatic aneurysms can be treated with liver resection, HAA ligation, or embolization as treatment options [5,10,11,12,13,14,15,16,17]. Currently, ruptured HAPs are frequently treated using an endovascular approach with the use of coiling and embolization, but standards for their management have not been established [5,7].

The aim of this review Is to present the current knowledge regarding the role of endovascular treatment of HAP after PD. This is a very important and clinically relevant topic since there are no other reviews summarizing various aspects of the risk factors and endovascular treatment of HAP after PD.

## 2. Clinical Importance of and Risk Factors for Post-Pancreaticoduodenectomy Pseudoaneurysms

Postoperative pseudoaneurysms, secondary to the local inflammatory process within the operative field, are one of the most common causes of postoperative bleeding following PD. The gastroduodenal artery, followed by the hepatic artery, is the most common location [2,18,19,20]. We can distinguish between early and late post-PD bleeding [2]. Early hemorrhages occur within the first 24 h after PD, and delayed post-PD hemorrhages occur between 24 h and 7 d following PD [2,21]. Post-PD bleeding caused by pseudoaneurysms is reported in 4–16% of cases. The mortality rate is high, at up to 50% in the following month [2,22]. There are various risk factors for post-PD pseudoaneurysms. Preoperative, intraoperative, and postoperative risk factors for developing a post-PD pseudoaneurysm can be distinguished. The preoperative risk factors include patient-related factors, such as a higher age, the male gender, a higher body mass index (BMI), comorbidities, and previous abdominal surgery. Intraoperative risk factors are associated with the duration of surgery, as well as the intraoperative surgical technique, including tissue dissection, lymph node removal, and resection and reconstruction of the blood vessels, as well as the type of pancreatic anastomosis. The tunica adventitia of the arterial wall can be damaged during tissue and lymph node dissection, and this can lead to developing a pseudoaneurysm. The use of electrotomes and ultrasound scalpels may damage the adventitia and cause pseudoaneurysm development during lymph node removal. Also, during tissue dissection, it is inevitable to clamp the tissue, leading to arterial wall damage [2,23,24,25,26]. Postoperative pseudoaneurysm risk factors are related to the postoperative conditions and complications, such as postoperative biliary drainage, as well as POPFs and postoperative biliary fistulae (POBFs) and post-pancreatectomy acute pancreatitis (PPAP). Therefore, other risk factors for the above-mentioned complications, including indications for PD (pancreatic metastasis and serous cystic neoplasms (SCNs) and neuroendocrine and ampullary neoplasms), as well as poor nutritional status, blood transfusion, blood loss, soft pancreatic texture, pancreatic lipomatosis, small pancreatic ducts (<3 mm) and tiny, thin-walled bile ducts (<5 mm), and bile infection, are also indirect risk factors for post-PD pseudoaneurysms. Pancreatic and biliary leakage, as well as inflammatory processes within the epigastrium, causes arterial wall erosion and extravasation of the blood, leading to the formation of a fibrotic capsule, which may be followed by a pseudoaneurysm rupture. Most frequently, pseudoaneurysms secondary to the above-mentioned factors are located within the gastroduodenal artery, followed by the hepatic, splenic, and intestinal branches of the superior mesenteric artery [1,2].

It Is Important to be aware of the above-mentioned risk factors for the development of post-PD pseudoaneurysms in order to prevent them and decrease their rates. Intraoperatively, the adequate surgical technique, as well as careful and solely necessary vessel dissection, is essential to decrease the risk of post-PD pseudoaneurysm. In addition, the prevention of postoperative complications, including POPFs, POBFs, and PPAP, is required to decrease the risk of post-PD pseudoaneurysms.

## 3. Clinical Manifestation and Diagnostics of Hepatic Artery Pseudoaneurysms

Currently, most cases of HAP are discovered incidentally during imaging investigation studies; however, in 80% of cases, aneurysm rupture is the first manifestation of HAP. Non-ruptured HAPs are usually asymptomatic [5,10,27]. Nausea and right upper quadrant pain radiating to the back are clinical HAP signs. When a pseudoaneurysm ruptures into the biliary tree, Quincke’s classic triad (jaundice, biliary colic, and gastrointestinal bleeding), as a manifestation of hemobilia, is observed. However, this triad is reported in only one-third of patients. According to the literature, 20–30% of HAPs rupture into the peritoneal cavity, leading to an intra-abdominal hemorrhage, with a high mortality rate (up to 82%) [5,10,28,29]. In the case of a ruptured HAP, the usual clinical presentation includes abdominal pain associated with distension and clinical signs of upper gastrointestinal bleeding (UGIB), including hematemesis, hematochezia, and melena once pseudoaneurysm rupture has occurred [2,3,30,31]. Due to the hemoperitoneum secondary to pseudoaneurysm rupture, signs of peritoneal irritation can be observed. Sentinel bleeding is usually a precursor of a massive hemorrhage [2,3]. Due to the typical clinical manifestation of UGIB, endoscopic examinations (panendoscopy and colonoscopy) are usually initially performed. However, they cannot show active hemorrhage or the bleeding source. Therefore, the preferred diagnostic method is computed tomography angiography (CTA), with a sensitivity rate of 95%. The practical possibility for its performance depends on the patient’s clinical condition and hemodynamic stability. Most patients with early hemorrhages are not fit for this investigation due to their critical and urgent condition [2,3]. Currently, selective celiac angiography is the gold-standard diagnostic and therapeutic management for postoperative bleeding and pseudoaneurysm in patients following PD. Although arteriography is an invasive procedure, it determines the exact bleeding and aneurysm location and aneurysm size, and it allows for immediate endovascular treatment. There are several conditions that can lead to false negative arteriography results, such as a distal location from the main branch and a slow flow rate of the contrast medium, in addition to the superimposition of intestinal gas and movement artifacts [2,3]. Generally, HAAs can be diagnosed by ultrasound examination, CTA, or digital subtraction angiography (DSA). According to *The Society for Vascular Surgery clinical practice guidelines on the management of visceral aneurysms*, CTA is the recommended diagnostic tool for HAAs (Grade 1B); meanwhile, mesenteric angiography for preoperative planning is recommended for patients with HAAs who are considered for intervention (Grade 1B) [10,32]. On the other hand, CTA is much more accurate for the identification of HAPs and their complications, and arteriography, as the gold diagnostic standard, allows for more exact HAP visualization and immediate endovascular therapeutic intervention [10]. In practice, CTA is initial imaging for diagnosis of the presence of HAP, and in patients with HAPs demonstrated in CTA, selective celiac arteriography is performed.

## 4. Management of Hepatic Artery Pseudoaneurysms According to the Society for Vascular Surgery on the Management of Visceral Aneurysms

Differential diagnosis of true and false HAAs is important to the choice of proper management. Surgery or trauma being in a patient’s medical history is essential in differential diagnosis. In radiological investigations, focal arterial disruption in the setting of otherwise normal arteries and inflammatory changes around an irregular aneurysm sac are useful for distinguishing HAPs from HAAs [5,10,32]. Moreover, in contrast to HAAs (which are asymptomatic), most HAPs are symptomatic, with their clinical signs including gastrointestinal bleeding or hemobilia [33]. According to the clinical practice guidelines of the Society for Vascular Surgery on the management of visceral aneurysms, all HAPs are associated with a high risk of rupture and significant mortality and therefore should be repaired as soon as a diagnosis is made regardless of cause (Grade 1A). Indications for intervention in true HAAs are more limited. Their repair is recommended in all symptomatic HAAs regardless of their size (Grade 1A); asymptomatic HAAs in patients without significant comorbidity; in HAAs with a diameter of >2 cm (Grade 1A) or if the aneurysm enlarges by >0.5 cm/y (Grade 1C); in HAAs >5 cm in patients with significant comorbidities (Grade 1B); and in all HAAs, regardless of size, in patients with vasculopathy or vasculitis (Grade 1C), as well as patients with HAAs with positive blood cultures (Grade 1C) [32,33].

Surgical and endovascular methods are used in HAP treatment. Surgery involves finding the parent artery of the pseudoaneurysm and ligating it with filaments or clamping it with titanium alloys. Endovascular treatment involves transarterial embolization (TAE), stent graft implantation, stent-assisted coiling, and balloon remodeling techniques [3,33]. The surgical goal is to remove the aneurysm and maintain hepatic circulation. An endovascular-first approach to all hepatic artery aneurysms is recommended if anatomically feasible (i.e., if this approach maintains arterial circulation to the liver) (Grade 1A) [32,33]. In patients with extrahepatic aneurysms, open and endovascular techniques are recommended to maintain liver circulation (Grade 1A) [32,33]. Coil embolization of the affected artery is recommended for patients with intrahepatic aneurysms (Grade 1B). Meanwhile, resection of the involved hepatic lobe is recommended for patients with large intrahepatic HAAs to avoid significant liver necrosis (Grade 1C) [32,33]. Open surgical repair or endovascular repair of visceral artery aneurysms yields similar long-term results, but the morbidity is significantly worse with open repair [33,34,35]. Therefore, endovascular techniques should be preferentially offered for anatomically suitable candidates. Overall, endovascular treatment has become the mainstream technique. However, open repair remains the therapeutic option with definite efficacy and is mostly chosen for HAA cases of a ruptured asymptomatic common hepatic artery (>2 cm) or an asymptomatic common hepatic artery in patients with fibromuscular dysplasia or polyarteritis nodosa and proper hepatic and proximal right or left hepatic branches [33,34,35]. The current recommendations for hepatic artery aneurysms and pseudoaneurysms are presented in Table 1.

## 5. Surgical versus Endovascular Treatment of Hepatic Artery Pseudoaneurysm

Surgical treatment of visceral pseudoaneurysms, including revascularization, artery ligation, or end-organ resection, is related to high (5–25%) mortality rates. The advantages of endovascular methods are higher safety and a less invasive approach compared to open surgery. The disadvantages of endovascular embolization are a high failure rate and exclusion of the distal circulation, which can lead to organ hypoperfusion and dysfunction. Stent graft exclusion of pseudoaneurysms is a promising alternative method preserving the blood flow within the artery and does not lead to complications secondary to organ hypoperfusion [9,36,37,38,39,40,41,42,43,44].

TAE’s success rate is 83–100%, with a mortality rate of 0–20%. Endovascular treatment has decreased patient mortality significantly, by up to 30%. According to the literature, its effectiveness for hemorrhage control is up to 95% [2]. Although it is a less invasive treatment, it is also associated with complications such as uncontrolled bleeding, failed coil embolization attempts, liver failure following embolization of the hepatic artery, liver abscesses, hepatic infarction without liver failure, and post embolization bacteremia in up to 20% of cases [2]. More commonly, transient signs of ischemia manifest with a higher serum activity of transaminases; less frequently, serious consequences of the occlusion of the end-organ vessels, leading to liver infarction and abscess formation, are reported [39]. It should be added that the liver can tolerate significant arterial embolization without significant adverse effects because of its multiple collateral pathways [36,45]. However, inadvertent occlusion of the wrong artery can lead to liver infarction and secondary liver abscesses as short-term postoperative complications [36]. The recurrence of pseudoaneurysms after successful embolization as a long-term complication has been also reported [39]. Following the implantation of stent grafts, stent occlusion, deformation, or kinking and the exclusion of branch vessels can be observed as potential complications [39].

The method of TAE depends on the location, size, and diameter of the major artery, as well as the presence of collateral branches to avoid complications related to organ infarction due to closing the blood supply vessels. The method of choice in most visceral aneurysms is the use of coils [2]. In pseudoaneurysms, liquid TAE is also performed (i.e., *N*-butyl-2-cyanoacrylate (*N*-BCA)). Stent graft implantation is the treatment of choice in patients with a favorable arterial anatomy (proximal artery pseudoaneurysms, adequate proximal and distal necks, a 5 to 10 mm artery length before and after the pseudoaneurysm, without arterial division and an adequate caliber, and a vessel pathway for performing safe catheter navigation), as well as with contraindications for TAE due to the high risk of organ hypoperfusion, particularly in patients with hepatic artery pseudoaneurysms and concomitant portal vein thrombosis. In these patients, TAE could lead to critical liver ischemia. Stent grafts are also recommended as the second-line management following technical TAE failure or recurrent bleeding secondary to embolization [9]. It should be pointed out that sepsis is not an absolute contraindication for stent graft implantation [9,46,47,48,49,50,51]. A literature review by Miller et al. [50] showed no cases of stent graft infection in the surgical outcomes [9]. According to Boufi et al. [9], prolonged systemic antibiotics administration can successfully prevent bacterial stent graft contamination [9]. In patients with preoperative sepsis, the authors recommend intravenous antibiotics to be systematically administered in the perioperative period, followed by prolonged (from 3 to 6 months) oral administration [9]. Moreover, stent graft implantation is successfully performed in patients with infected pseudoaneurysms and mycotic aneurysms, as well as aortoesophageal fistulae. All the above-mentioned diseases are associated with inflammatory processes and infection [9,52,53,54,55,56,57,58].

Both balloon-expandable stent grafts with the over-the-wire technique and self-expandable stent grafts are used. Self-expandable stent grafts are useful in tortuous and small arteries [9]. It has been shown that stent graft implantation enables fast and efficient bleeding control in hemodynamically unstable patients [51]. Stent graft implantation in hemodynamically unstable patients is performed by operating on the patient under general anesthesia. Stent graft implantation in hemodynamically stable patients is performed in an angiographic room with local anesthesia and conscious sedation. These procedures are performed using femoral or brachial access [9].

**Figure 1:**
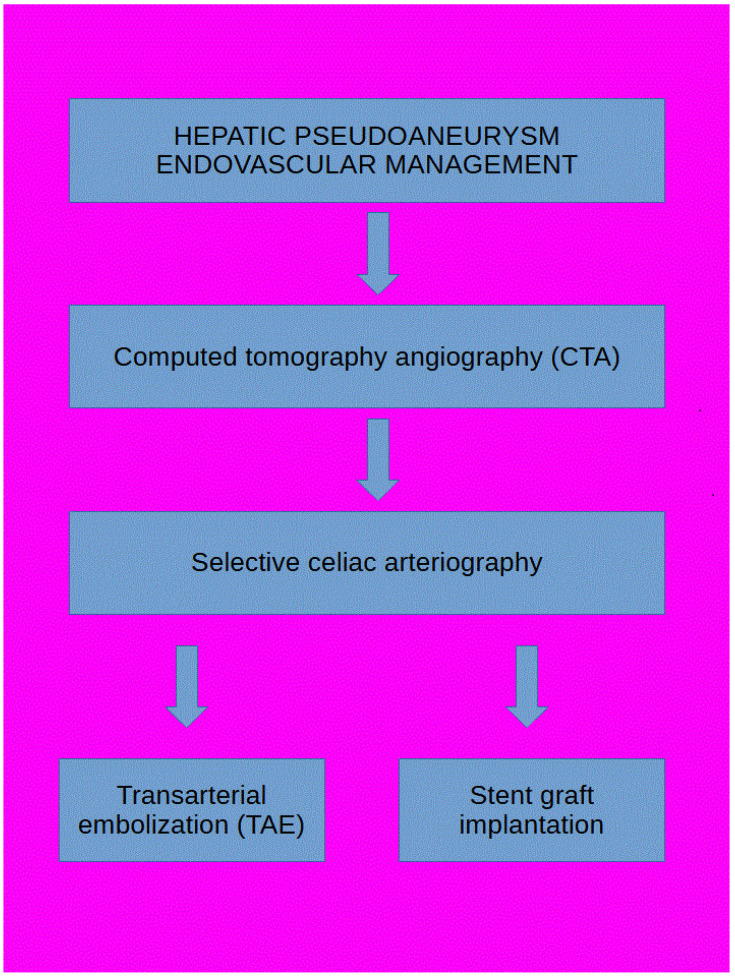
Algorithm for endovascular management of hepatic artery pseudoaneurysms.

An algorithm for endovascular management of hepatic artery pseudoaneurysms is presented in Figure 1.

## 6. Technical Aspects of Endovascular Treatment of Hepatic Artery Pseudoaneurysms

Endovascular access using the Seldinger technique for endovascular procedures is performed most frequently through percutaneous puncture of the femoral artery, followed by the brachial artery. Next, a 5 F or 6 F sheath is usually inserted, and next, a shaped guiding catheter is inserted to selectively gain access to the celiac trunk and the hepatic artery. Before selective arteriography, first, abdominal aortography using a pigtail catheter is performed to find the ostium of the celiac axis. Subsequently, the artery is selectively catheterized, and pre-treatment selective arteriography is performed to measure the pseudoaneurysm. Sometimes, intravascular ultrasound (IVUS) is required to assist in the identification of a pseudoaneurysm which is not clearly shown in selective arteriography. Next, during the TAE procedure, a 2.5 F microcatheter is inserted into the target artery using a coaxial catheter technique for super-selective arteriography and embolization. In others, a triaxial endovascular system with a 3 F microcatheter is used in most interventions for the selective embolization of a pseudoaneurysm cavity with preservation of the proximal parent artery [9,59].

Various TAE techniques are described: using intravascular coils, gelatine foam, cyanoacrylate glue, ethanol sclerosant, and detachable balloons [60,61]. The common methods of pseudoaneurysm TAE involve simple lumen embolization (the sac packing technique), proximal embolization of the parent artery (the proximal embolization technique), inflow and outflow embolization of the parent artery (the exclusion technique or the isolation technique), and efferent artery embolization + sac packing/aneurysmal neck packing + afferent artery embolization (the sandwich technique). Sac packing is performed for saccular pseudoaneurysms with a narrow neck, which allows for the retention of coils within the sac, maintaining the patency of the parent artery. Proximal embolization of the parent artery is used in pseudoaneurysms at the end of arterioles, including or excluding sac packing, which is a special exclusion technique. The exclusion technique is performed in pseudoaneurysms with a small diameter, a wide neck, and a short landing zone, which refers to the area of proximal and distal stent placement and vascular remodeling. The sandwich technique is performed for pseudoaneurysms with collateral inflow and outflow arteries [3]. In stent graft implantation, a balloon-expandable or self-expanding nitinol stent is selectively inserted to preserve flow within a hepatic artery in order to prevent postoperative liver ischemia and ischemia-related secondary post-embolization complications (liver necrosis, infection, infarction, and abscesses). Following TAE or stent graft implantation, control arteriography is performed to assess the effect of the procedure and to confirm the exclusion of the pseudoaneurysm. Following control arteriography, the whole system is removed from the artery. The site of endovascular access is closed or/and a sterile compression dressing is applied [3,9,59].

According to the literature, the success rate of TAE for visceral artery pseudoaneurysms is 63–100%, with a morbidity rate of 14–25% and a mortality rate of 0–14%. In up to 37% of patients, recanalization or rebleeding is reported [60,61,62,63].

**Figure 2:**
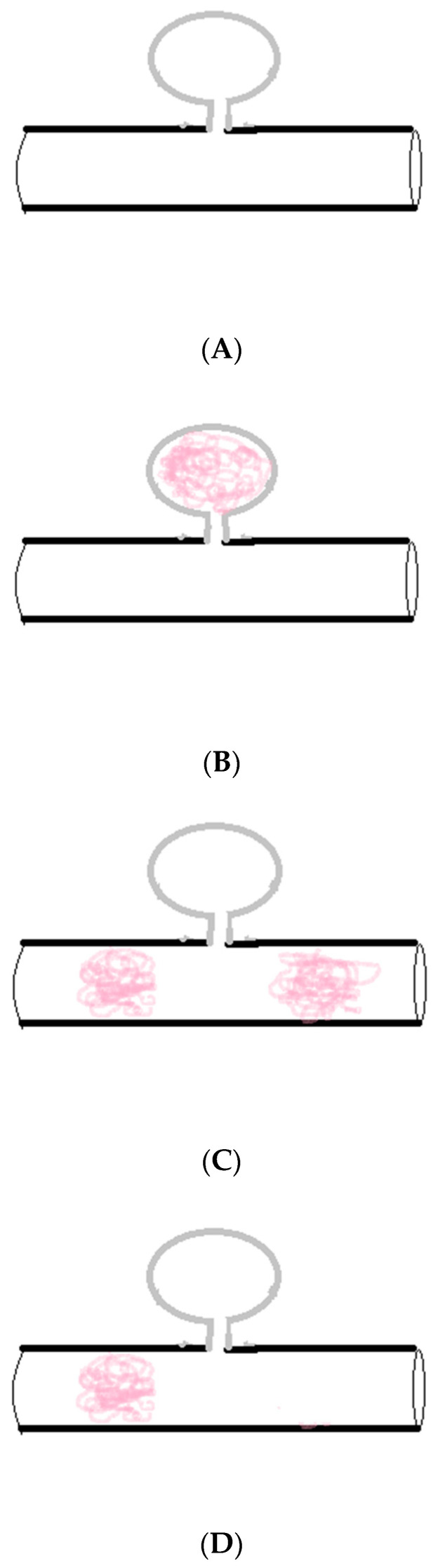
(**A**) Pseudoaneurysm with a narrow neck. (**B**) **Sac packing**. This technique involves filling the pseudoaneurysm with coils or microcoils, typically using a coaxial technique. It is performed for saccular pseudoaneurysms with a narrow neck, allowing for retention of coils within the sac, maintaining the patency of the parent artery. (**C**) **Proximal and distal packing (sandwich technique).** This is performed for pseudoaneurysms with collateral inflow vessels. Occlusion is performed distal to, across, and proximal to the neck of the pseudoaneurysm, blocking the efferent (back door) and afferent arteries (front door). Embolization of only the parent or afferent artery could lead to incomplete embolization and recurrence due to retrograde filling from the efferent collateral. The efferent artery or back door is closed first, followed by the afferent artery or front door. (**D**) **Proximal packing.** This is performed for end arteries (without collateral inflow vessels) in which it is sufficient.

**Figure 3:**
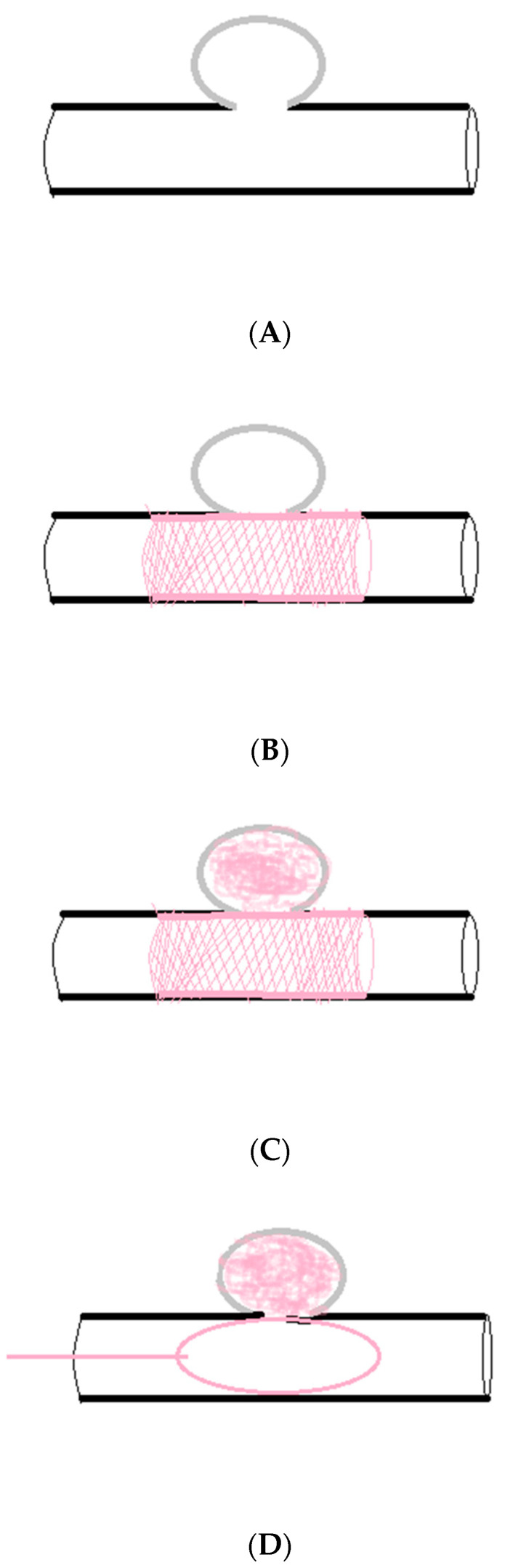
(**A**) **Pseudoaneurysm with a wide neck/fusiform.** These are characterized by an increased risk of migration of embolic material. (**B**) **Stent graft implantation.** This technique preserves the patency of the parent artery. It is performed for larger proximal arterial segments like the common/proper hepatic artery. (**C**) **Stent-assisted coiling.** This is performed in cases where the parent artery is inexpandable in order to prevent the coils from projecting into the lumen. The bare stent is implanted across the neck of the pseudoaneurysm. It acts as a scaffold for coil embolization through the gaps in the stent. (**D**) **Balloon-assisted coiling.** This is performed in cases where the parent artery is inexpandable in order to prevent the coils from projecting into the lumen. The balloon catheter is inserted across the neck of the pseudoaneurysm. It acts as a scaffold for coil embolization to the side of balloon.

A summary of various techniques for endovascular treatment of visceral aneurysms, including HAPs, depending on the aneurysm location, size, and shape, as well as the presence of collateral vessels, is presented in Figure 2 and Figure 3 [64,65].

## 7. A Literature Review of Case Reports and Case Series on Endovascular Treatment of Hepatic Artery Pseudoaneurysms

### 7.1. Methods for the Literature Search

A literature search was performed using the PubMed database. The search strategy was “hepatic artery” AND “pseudoaneurysm” AND “pancreaticoduodenectomy”. Initially, full-text articles published in 2000–2024 were found (n = 62). The inclusion criteria included HAP treated using endovascular methods (TAE or stent graft) developed directly after PD. Case reports and case series including information on the patient’s age, gender, clinical manifestation, HAP location, type of endovascular procedure, and outcome were included in the further analysis. Cases with true aneurysms, aneurysms following reoperations after PD, aneurysms involving a gastroduodenal artery stump, and open surgical treatment and articles with non-granular pooled data were excluded. Articles with incomplete information regarding all the treated patients (including the clinical manifestation) were excluded from further analysis. In addition, a case report presenting a pseudoaneurysm within the CHA manifested and treated 10 months following total pancreatectomy was excluded from the analysis. Finally, 12 articles (7 case reports, 2 case series, and 3 retrospective studies) were included in our analysis. Cases were assessed according to demographics, clinical presentation, pseudoaneurysm site, and management technique. Therefore, finally, our analysis included three articles (two case reports and one case series) involving 30 cases of symptomatic bleeding HAPs.

### 7.2. A Short Description of Case Reports and Case Series on Endovascular Treatment of Hepatic Artery Pseudoaneurysms

Recently, Ayala et al. [2] described a 62-year-old female patient with a pseudoaneurysm located within the PHA manifesting as gastrointestinal bleeding and intra-abdominal hemorrhage with secondary hypovolemic shock following PD for a periampullary tumor. Before bleeding, intra-abdominal fluid collection was drained. In this patient, endovascular treatment with common hepatic artery embolization using coils was performed, with successful bleeding control. 

Kawa et al. [39] reported a 62-year-old male patient with a pseudoaneurysm located within the right hepatic artery manifesting as upper gastrointestinal bleeding (melena and lightheadedness) and anemia following PD for cholangiocarcinoma. Before bleeding, intra-abdominal fluid collection was conservatively (antibiotics) treated.

Tanaka et al. [63] described a 74-year-old male patient with a pseudoaneurysm located within the CHA with no active bleeding following total pancreatectomy for pancreatic cancer, with no clinical signs initially, followed by upper gastrointestinal bleeding (melena) and hypovolemic shock. Two balloon-expandable stent grafts were inserted into the CHA. After good short-term results, one month later, melena and hemodynamic shock presented, and a pseudoaneurysm within the bifurcation of the CHA and the PHA was demonstrated in arteriography. Then, TAE using micro coils (3 TRUFILL coil, Cordis Endovascular Systems, Johnson & Johnson) was performed. Additional coiling was performed in the PHA. The blood flow via the hepatic artery was blocked by microcoil embolization (10 TRUFILL coils and 6 Diamond coils; Cordis Endovascular Systems, Johnson & Johnson) in the lumen of the stent graft due to the vulnerable PHA and a high risk of rebleeding. Control arteriography showed complete exclusion of the CHA and cessation of bleeding and blood flow to the liver via the anastomotic branch of the left gastric artery (accessory left gastric artery). Therefore, liver ischemia was not reported in this case. There were no other complications or rebleeding within 18 months.

Harvey et al. [66] described a 61-year-old man with von Willebrand’s syndrome who had undergone PD for a distal common bile duct tumor. Seven days following surgery, upper gastrointestinal bleeding leading to hypovolemic shock was recorded, and emergency arteriography visualized a pseudoaneurysm within the CHA. The pseudoaneurysm was managed with a 6 mm Viabahn stent graft. Distal migration of the stent graft was noted following this procedure. Therefore, a second procedure was performed to stabilize the first and exclude the pseudoaneurysm [66].

Sasaki et al. [67] described the case of a 73-year-old man following PD for distal cholangiocarcinoma. A POPF, initially treated conservatively, was reported after surgery. On postoperative day (POD) 86, blood loss of 100 mL from the POPF site was noted. A pseudoaneurysm within the CHA (12 mm of diameter) was shown in CECT. The diameter of the pseudoaneurysm increased to 15 mm on the 89th postoperative day. Thus, coronary covered stents were implanted to prevent massive bleeding secondary to its rupture and to retain hepatic arterial flow. CT confirmed a thrombosed pseudoaneurysm and proper hepatic arterial flow. The post-procedure course ensued without any complications [67].

Hankins et al. [68] reported a case of an HAP within the PHA in a 51-year-old man following PD for pancreatic adenocarcinoma. On postoperative day 26, tachycardia with sentinel bleeding around the postoperative drains was noted. The first hepatic angiography did not show an active hemorrhage or a pseudoaneurysm. On postoperative day 40, tachycardia and hypotension secondary to recurrent bleeding developed. The second hepatic angiography revealed a pseudoaneurysm within the PHA with active bleeding. Thus, a stent graft was implanted without any post-procedure complications [68].

Asai et al. [69] presented the case of a CHA pseudoaneurysm secondary to a POPF after PD in a 70-year-old female who had undergone PD for middle cholangiocarcinoma. The POPF was reported on postoperative day 7, and blood loss of 500 mL via the abdominal drain on POD 19 was noted. Urgent celiac arteriography revealed a CHA pseudoaneurysm, which was treated using a coronary covered stent without any complications [69].

A study by Stoupis et al. [51] presented a case series of five male patients aged 50–76 years old with CHA HAPs following PD for chronic pancreatitis (n = 3), pancreatic adenocarcinoma (n = 1), and a duodenal diverticulum (n = 1) treated using stent grafts. Postoperative bleeding was reported on PODs 16–32. Three patients died due to abdominal sepsis (n = 1), multiorgan failure (n = 1), and neuromyopathy and renal failure (n = 1), and two patients survived in this group [51].

A retrospective study by Herzog et al. [70] involved three male patients aged 58–79 years old with RHA HAPs following PD for chronic pancreatitis (n = 1), pancreatic cancer (n = 1), and distal bile duct cancer (n = 1). All these patients manifested visceral bleeding and secondary anemia on PODs 15–36, and HAPs in all of them were treated successfully using stent grafts with no complications. In all three patients, bacteriobilia was reported [70].

Heiss et al. [71] described a case series of four male patients with delayed post-PD hemorrhages, including two patients aged 48–56 years old with post-PD HAPs located within the CHA and the PHA, managed using stent grafts. PD was performed for adenocarcinoma of the papilla of Vater and chronic pancreatitis with suspicion of carcinoma of the pancreatic head. In both patients, the HAPs manifested as active visceral hemorrhages presenting 1, 3, and 5 weeks after PD in the first patient and 4 weeks after PD in the second patient. In both patients, delayed visceral arterial hemorrhage was diagnosed angiographically and treated endovascularly using stent grafts with no complications [71].

**Figure 4:**
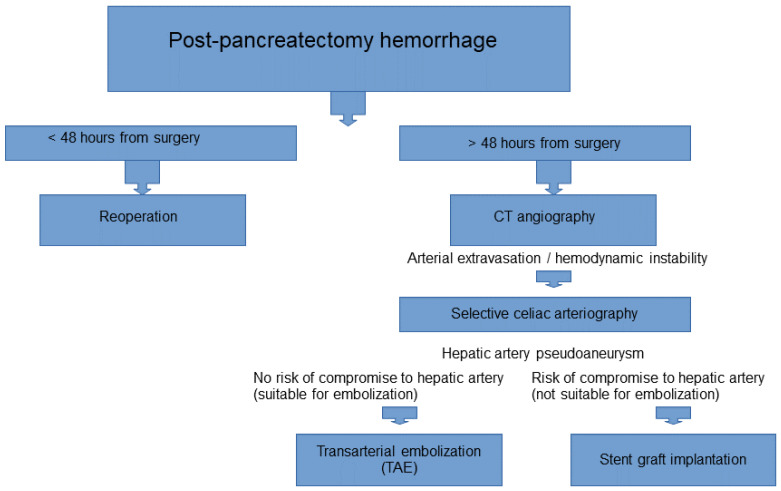
Algorithm for management of post-pancreatectomy hemorrhage.

Wang et al. [72] presented a series of nine cases of life-threatening hemorrhage due to a ruptured HAP within the CHA following PD for pancreatic cancer (n = 6), distal common bile duct cancer (n = 1), periampullary cancer (n = 1), and pancreatic trauma (n = 1), treated using stent grafts. This study included six men and three women aged 23–75 years old (mean age = 48 years). The clinical manifestation of the HAP bleeding involved bleeding from the abdominal drain (n = 7), hematemesis (n = 3), melena (n = 1), and blood in the nasogastric tube (n = 2). In this study, in all the patients, embolization was not possible because of a non-patent portal vein. There were no early post-procedure complications, and implantation of a stent graft was successful in all patients. Recurrent bleeding was noted in two patients at 16 and 24 h, respectively. It was treated with surgical revision. Three patients died due to recurrent uncontrolled bleeding (n = 1), multiorgan failure (n = 1), and abdominal sepsis (n = 1) after the stent graft implantation. Based on the above-mentioned results, the authors concluded that stent graft implantation is an effective and safe procedure for acute life-threatening hemorrhage from ruptured HAPs. The authors pointed out several limitations of stent graft implantation post-HAP. Stent graft implantation into the branches of the celiac trunk is not always technically possible. The procedure may lead to artery rupture because of its eroded and fragile vascular wall, thus requiring emergency vascular surgery. Finally, stent graft implantation may lead to in-stent-graft stenosis and occlusion. Therefore, antiplatelet medication is recommended after stent graft deployment in order to prevent in-stent-graft stenosis [72]. According to Finch et al. [73], in patients with post-PD HAPs, in whom embolization is associated with a risk of occlusion with compromise of the liver’s arterial inflow, stent graft implantation is an important hemostatic option but is associated with a high risk of subsequent graft occlusion. Their retrospective study involved 440 patients undergoing PD. Sixty-seven (15%) experienced postoperative hemorrhage. POPFs were significantly more frequent in the postoperative hemorrhage group, which confirms that POPFs are an important and common cause of post-PD bleeding. In the bleeding patients, the following interventions were performed: reoperation in 15 (22%), embolization in 9 (13%), and stenting in 7 (10%). There were three patients with HAPs treated using stent grafts. HAPs were located within the proximal CHA (n = 2) and the distal CHA and proximal PHA (n = 1). Recurrent bleeding was reported in HAPs within the distal CHA and PHA, and it required reoperation and embolization. A hepatic abscess was noted in one patient following stent graft implantation into the HAP located within the proximal CHA [73]. Finch et al. proposed an algorithm for the use of hepatic artery endovascular stents in post-pancreatectomy hemorrhage (Figure 4).

In a retrospective study by Boufi et al. [9], endovascular treatment using stent graft implantation was analyzed in 10 patients with visceral artery aneurysms. There were eight patients with HAPs following PD in the analyzed group. The patients presented a wide spectrum of clinical signs, from no symptoms, upper gastrointestinal bleeding (hematemesis, melena), abdominal pain, fever, a pulsatile mass in the right upper abdomen, and bleeding through abdominal drains to hypovolemic shock and sepsis. POPFs were confirmed in two patients. In most patients, hemodynamic instability was observed. There were no postoperative complications related to stent graft implantation in most of the patients. One patient died due to multiorgan failure following relaparotomy [9].

In a retrospective study by Cui et al. [26], 17 patients undergoing stent graft implantation due to HAPs were analyzed. There were 11 post-PD patients in this group. Ultimately, four of these patients died due to multiple organ failure within 2–10 days. One to three stent grafts were implanted. Stable hemodynamics was achieved in 12 patients (stent diameter: 4.5–8 mm). In four patients (stent diameter: 3–4.5 mm), bleeding recurrence (1 h to 3 days after stent implantation) and type 1 endoleaks were identified during second arteriography. In three of them, coil embolization (entire hepatic artery embolization) was performed. Ultimately, these four patients died of multiple organ failure 2–10 days after embolization/blockage. In one patient, in the following 2 weeks, recurrent bleeding from the SMA was noted. The authors did not link postoperative outcome to the cause of HAP. Therefore, it can not be concluded what the surgical outcome was in patients with HAPs following PD [26]. All above described cases are summarized in Table 2. 

### 7.3. Results of the Summary Analysis of Case Reports and Case Series on Endovascular Treatment of Hepatic Artery Pseudoaneurysms

This analysis showed that HAP was noted most frequently in patients aged 59.92 (23–79) years old. HAP was more frequently reported in men compared to women (73.68% vs. 26.32%, respectively). Most frequently, HAP manifested on the 20th postoperative day (mean value: 20.41 (6–86)). The common hepatic artery was the most common HAP site (n = 24, 80%), followed by the right hepatic artery (n = 4, 13.33%) and the proper hepatic artery (n = 2, 6.67%). In 53.33% of patients (n = 16), bleeding through the postoperative abdominal drains was observed, and the analysis revealed that it was the most frequent clinical symptom of HAP bleeding. The second most common clinical sign was bleeding from the upper gastrointestinal tract (including hematemesis/melena/bleeding through a nasogastric tube), which was reported in 43.33% (n = 14) of patients. Hypovolemic shock was noted in 30% of patients (n = 9), while anemia was reported in 20% of patients (n = 6). The other clinical symptoms included abdominal pain (n = 2, 6.67%), fever (n = 1, 3.33%), and a palpable abdominal pulsative mass (n = 1, 3.33%). Stent graft implantation was the most frequent method of endovascular HAP treatment (96.67%, n = 29). TAE was performed as an initial endovascular treatment in only one (3.33%) patient. The need to maintain the flow to the liver is why stent grafts are superior to embolization. Post-procedure complications were noted in 30% of patients (n = 10). They included as follows: recurrent bleeding (n = 3, 10%), multiorgan failure (MOF) (n= 3, 10%), hepatic abscesses (n = 2, 6.67%), transient transaminase elevation (n = 1, 3.33%), distal stent migration (n = 1, 3.33%), and abdominal sepsis (n = 1, 3.33%). The post-procedure mortality was 16.17% (n = 5).

A summary of the most precisely documented endovascular treatment of HAPs following PD is presented in Table 2.

### 7.4. Discussion and Conclusions

Our analysis revealed that most frequently, HAPs are located within the CHA. They are usually related to other post-PD complications, such as POPFs and other infectious complications. They can be massive and even fatal. They are commonly manifested as bleeding through abdominal drains and upper gastrointestinal bleeding. HAP bleeding is a life-threatening and serious condition, leading to hypovolemic shock and anemia. Therefore, it needs emergency treatment. In patients with delayed post-PD hemorrhage, first, CECT should be performed. In patients with bleeding in CT, it should be followed by a selective celiac arteriography, which allows it to be visualized and bleeding to be stopped. Traditional surgical approaches (relaparotomy) in hemodynamically unstable patients are very difficult and challenging. Surgical re-exploration may be hazardous due to tissue friability, and identification of the bleeding site may be extremely difficult due to postoperative inflammatory infiltration and adhesions. Therefore, endovascular methods, which do not require relaparotomy, dissection, or looking for a bleeding site in a very difficult operative field, are very attractive alternative methods to surgery. The aim of endovascular treatment is to exclude a pseudoaneurysm from circulation and to stop bleeding. There are two main endovascular methods for excluding HAPs from circulation: TAE and stent graft implantation. It has been reported that stent graft implantation is the most frequently method used to treat HAP. It is associated with the need to preserve blood flow within the hepatic artery in order to prevent liver ischemia following this procedure. It is very important to exclude a pseudoaneurysm from circulation while maintaining blood supply to the liver. This is most important in patients with concomitant portal vein stenosis, in whom the risk of post-procedure liver ischemia is significantly higher. Therefore, stent graft implantation preserving vessel patency should be preferred over embolization if it is technically feasible. Generally, stent graft implantation is superior to TAE, but it does have some limitations. Wang et al. [72] presented limitations of stent graft implantation. Due to the difficult anatomy and tortuous arteries, stent graft implantation in the branches of the celiac trunk is not always technically possible. Stent graft implantation can lead to artery rupture because of an eroded and fragile vascular wall, thus requiring emergency vascular surgery. In-stent-graft stenosis and occlusion due to stent graft thrombosis is another stent-graft-related complication. Therefore, antiplatelet medication is recommended after stent graft deployment in order to prevent in-stent-graft stenosis [72]. Another difficulty associated with stent graft implantation is the selection of the proper stent graft size. The selection of the proper stent size (including diameter and length) can be challenging. Because the diameter of the affected vessel is often decreased as a result of hemodynamic instability and secondary vascular spasms, it is thus important to avoid undersizing to provide the appropriate sealing and avoid endoleaks and stent migration. On the other side, oversizing can lead to vessel rupture and stent graft thrombosis. Determination of the correct stent graft length is very difficult due to vascular spasms potentially disguising the entire extent of artery erosion. Especially in patients with arterial wall erosion caused by pancreatic or anastomotic leakage, finding the proper stent length with safe proximal and distal landing zones is important in order to avoid recurrent bleeding. The choice of the proper stent graft size should be based on angiographic findings and pre-interventional CECT. Hassold et al. prefer TAE (instead of stent graft implantation) in all patients with significant spasms within an affected artery, in whom stent graft implantation can be extremely hazardous and dangerous due to the very high risk of rupture of a very fragile artery [74].

Although endovascular treatment methods are less invasive percutaneous procedures, possible in most patients via femoral artery access, and do not require laparotomy, they are related to the risk of postoperative morbidity and mortality. Post-procedure morbidity involves hepatic ischemia/hepatic abscesses or transient transaminase elevation secondary to a disturbed blood flow to the liver, as well as stent graft or coil migration, stent graft occlusion, and infection. The most serious complications involve multiorgan failure and sepsis. Post-procedure mortality is related to a very serious patient condition, abdominal sepsis, multiorgan failure, hypovolemic shock, and post-procedure complications. It should be pointed out that some patients who experience post-procedure complications require emergency vascular surgery. Therefore, both TAE and stent graft implantation should be performed in centers with vascular surgery facilities. Although the postoperative morbidity rate following endovascular treatment is critical according to the literature, it is significantly lower compared to that following surgery. The above-mentioned analysis revealed 30% morbidity and 16% mortality following endovascular treatment. These results are similar to those in the literature. According to a meta-analysis by Limongelli et al. [75] comparing endovascular treatment and surgery in the management of delayed post-PD hemorrhage, the morbidity and mortality rates are as follows: morbidity rate (endovascular, 36%; surgery, 70%) and mortality rate (endovascular, 21%; surgery, 43%) [69,75]. Therefore, endovascular therapy is now considered to be the standard surgical management for delayed post-PD hemorrhage [69]. Thus, surgery is currently only recommended for those patients who cannot be resuscitated for endovascular treatment or in whom endovascular therapy has failed [76].

## 8. Summary and Conclusions

HAP is a serious complication of PD secondary to postoperative complications such as POPFs, POBFs, and POAP, as well as intraoperative extensive tissue dissection and lymphadenectomy. Both of the former can lead to arterial wall damage according to inflammatory processes and pancreatic juice digestion or direct arterial wall injury. HAP can be asymptomatic. Upper gastrointestinal bleeding (hematemesis and melena) and intra-abdominal hemorrhage (abdominal pain and distension) are the most common clinical manifestations of HAP. HAP rupture can lead to hypovolemic shock and hemodynamic instability in patients. It is associated with high mortality. Therefore, HAP requires treatment. Currently, selective celiac angiography is the gold-standard form of diagnostic and therapeutic management for postoperative bleeding and pseudoaneurysms in patients following PD. Open surgery and less invasive endovascular treatment are performed in patients with HAP. Endovascular treatment involves TAE and stent graft implantation. The choice of treatment method depends on the general and local conditions, such as the patient’s hemodynamic stability and arterial anatomy. In patients in whom preservation of the blood flow within the hepatic artery (to prevent hepatic ischemia complications such as liver infarction, abscesses, or failure) is needed, stent graft implantation is the treatment of choice because it allows the exclusion of the pseudoaneurysm from circulation, preservation of the blood flow within the hepatic artery, and avoidance of hepatic ischemia.

## Figures and Tables

**Table 1:** Current recommendations for hepatic artery aneurysms and pseudoaneurysms according to the Society for Vascular Surgery on the management of visceral aneurysms.

Recommendation		Strength of Recommendation	Quality of Evidence
2.1.	Due to the high risk of rupture and significant mortality, all hepatic artery pseudoaneurysms, regardless of cause, are recommended to be repaired as soon as a diagnosis is made.	1 (Strong)	A (High)
2.2.a	Repair of all symptomatic HAAs regardless of size is recommended.	1 (Strong)	A (High)
2.2.b	In asymptomatic patients without significant comorbidity, repair is recommended in the following cases: True HAAs >2 cm (Grade 1A) Aneurysms enlarging by >0.5 cm/y (Grade 1C). In patients with significant comorbidities, open repair is recommended as follows: In HAAs >5.0 cm (Grade 1B).	1 (Strong)	A (High)
2.3.	Repair of HAAs in patients with vasculopathy or vasculitis is recommended, regardless of size (Grade 1C). Repair in HAA patients with positive blood cultures is recommended (Grade 1C).	1 (Strong)	C (Low)

HAA, hepatic artery aneurysm.

**Table 2:** Summary of the most precisely documented endovascular treatments for hepatic artery pseudoaneurysms following pancreaticoduodenectomy.

Authors (Year)	Study Design	Postoperative Day	Clinical Manifestation	Location of Pseudoaneurysm	Treatment Method	Outcome
Ayala et al. (2023) [2]	Case report F 62	20	Upper gastrointestinal bleeding, hematemesis, and melena, 2 g/dL decrease in hemoglobin levels, hypovolemic shock	Proper hepatic artery	TAE with interlock coils	Success and no complications in 90 days
Kaw et al. (2006) [39]	Case report M 62	21	Upper gastrointestinal bleeding: melena and lightheadedness. Decrease in hemoglobin levels (8.6 g/dL).	Right hepatic artery	Stent graft	Success Complication: liver abscess in the left and caudate lobes 5 weeks after discharge, successfully treated with percutaneous catheter drainage
Tanaka et al. (2010) [63]	Case report M 74	10th month	Initially: no signs; after 7 days: upper gastrointestinal bleeding, melena, hypovolemic shock	Common hepatic artery	First: two stentgrafts Second: TAE with microcoil embolization	Pseudoaneurysm in the CHA bifurcation following first procedure. No complications following second procedure
Harvey et al. (2006) [66]	Case report M 61	7	Upper gastrointestinal bleeding, melena, hypovolemic shock	Common hepatic artery	Stent graft Second device to stabilize the first and exclude the pseudoaneurysm	Distal migration of stent graft requiring second device Transient mild elevation in serum transaminases but no evidence of hepatic insufficiency or ischemia following second procedure
Sasaki et al. (2009) [67]	Case report M 73	86	Blood loss from the site of the pancreatic fistula	Common hepatic artery	Stent graft	No complications
Hankins et al. (2009) [68]	Case report M 51	26 (40)	26th day: tachycardia, bleeding around the postoperative drains; 40th day: hypovolemic shock	Common hepatic artery	Stent graft	No complications
Asai et al. (2011) [69]	Case report F 70	19	Bleeding via postoperative drains	Common hepatic artery	Stent graft	No complications
Herzog et al. (2011) [70]	A retrospective study including a case series of three patients	15–36	Visceral bleeding, anemia	Right hepatic artery	Stent graft	No post-procedure complications Bacteriobilia
	M 58	15				
	M 58	11				
	M 79	36				
Heiss et al. (2007) [71]	Case series	7–28	Visceral bleeding	Common hepatic artery Proper hepatic artery	Stent graft	No complications
	M 56	7				
	M 48	28				
Finch et al. (2011) [73]	A retrospective study including three patients with HAPs treated with stent grafts No data No data No data	No data	No data	Common hepatic artery	Stent graft	No complications Recurrent bleeding Hepatic abscess
Wang et al. (2010) [72]	Case series	6–38		Common hepatic artery	Stent graft	
	F 75	14	Abdominal drain			No complications
	F 23	9	Abdominal drain			No complications
	M 42	15	Abdominal drain			No complications
	M 56	7	Abdominal drain, hematemesis			Recurrent bleeding, death
	M 62	35	Hematemesis, melena			No complications
	M 67	6	Abdominal drain, nasogastric tube			No complications
	M 53	38	Hematemesis, melena			No complications
	M 68	8	Abdominal drain,			No complications
	F 50	6	Abdominal drain, nasogastric tube			Recurrent bleeding, MOF, death Abdominal sepsis, death
Boufi et al. (2011) [9]	A retrospective study on 10 patients, including 8 patients after PD	7–29,		Common hepatic artery	Stent graft	
	71	29	Abdominal pain, melena			No complications
	52	16	Abdominal drain, upper gastrointestinal bleeding			MOF, death
	65	22	Upper gastrointestinal bleeding, hypovolemic shock			No complications
	47	27	Anemia, abdominal pain, Abdominal pulsative mass			No complications
	76	18	Upper gastrointestinal bleeding, melena, hypovolemic shock			MOF, death
	75	7	Abdominal drain, melena, hypovolemic shock			No complications
	56	24	Fever, melena, hypovolemic shock			No complications
	53	20	Upper gastrointestinal bleeding, hypovolemic shock			No complications

F, female, M, male; PD, pancreaticoduodenectomy; CHA, common hepatic artery, TAE, transarterial embolization; MOF, multiorgan failure.

## Data Availability

No new data were created or analyzed in this study. Data sharing is not applicable to this article.
